# Landscape and local site variables differentially influence pollinators and pollination services in urban agricultural sites

**DOI:** 10.1371/journal.pone.0212034

**Published:** 2019-02-13

**Authors:** Ashley B. Bennett, Sarah Lovell

**Affiliations:** Department of Crop Sciences, University of Illinois, Urbana, Illinois, United States of America; Chinese Academy of Forestry, CHINA

## Abstract

Urbanization has detrimental effects on biodiversity and ecosystem functioning, as agricultural and semi-natural habitats are converted into landscapes dominated by built features. Urban agricultural sites are a growing component of urban landscapes and have potential to serve as a source of biodiversity conservation and ecosystem service provisioning in urban areas. In 19 urban agricultural sites, we investigated how surrounding land cover and local site variables supported bees and pollination services. We found the abundance of bees differentially responded to landscape and local scale variables depending on body size and nesting habit. Large-bodied bees, *Bombus* and *Apis* species, were positively associated with increasing amounts of impervious cover, while the abundance of small-bodied soil nesting *Halictus* species increased as the proportion of flower area, a local variable, increased. Bee richness declined with increasing levels of impervious cover, while bee community composition changed along a gradient of increasing impervious cover. Pollination services, measured at each site using sentinel cucumber plants, declined as hardscape, a local variable, increased. To improve bee conservation and pollination services in urban agricultural sites, our results suggest urban planning strategies should minimize impervious cover at large spatial scales while land managers should focus locally on incorporating floral resources, which increases food and nesting resources especially for smaller bee species. Local site design coupled with regional urban planning can advance the success of urban agriculture, while benefiting biodiversity by creating opportunities for pollinator conservation in urban landscapes.

## Introduction

Urbanization displaces agricultural and semi-natural landscapes, threatening the diversity of existing plant and animal species [1]. However, urban planning strategies can be developed and implemented based on conservation goals to reduce the loss of biodiversity [2–6]. Urban areas are increasingly recognized for their potential to support a broad diversity of species, and advocates for urban conservation suggest urban green spaces in particular can play an important role in biodiversity conservation [7]. As a result, greater emphasis is being placed on the value of urban green spaces to conserve biodiversity and restore ecosystem services that are lost as the built environment grows [3, 8–9].

The term urban green space is used to represent the private and public spaces of a city, often containing a variety of habitats and supporting multiple uses [10]. Many different types of land contribute to urban green space including natural areas, neighborhood parks, boulevards, golf courses, and residential yards, all of which have the potential to support urban biodiversity [11]. One type of green space that is expanding in many cities, in part due to the availability of vacant lots and demand for locally grown food, is urban agriculture. The production of food in cities occurs in urban farms, community gardens, backyard gardens, and green roofs. While research into the ability of urban green spaces to support biodiversity is growing [12–14], current understanding around the capability of urban agriculture to support biodiversity is limited [15–16]. Urban agricultural sites, like other types of green space, hold promise as sources of urban biodiversity conservation because they support a broad range of plant species, which in turn support biodiversity at upper trophic levels capable of providing ecosystem services.

Pollination of food crops by bees has been a topic of growing interest and increasing concern with declining populations of wild bees [17–18]. In rural agricultural settings, pollination of fruit-producing crops is often supplemented by placing managed bees such as honey bees (*Apis mellifera*) in fields. While honey bees are common in some cities that allow colonies for honey production and these bees may contribute to pollination, crop pollination in urban agricultural sites is likely to also rely on wild bees [19–20]. In New York City community gardens, for example, the common eastern bumble bee (*Bombus impatiens*) was found to be the most abundant native bee, and the only bee species found at all sites sampled [19]. Declining bee populations worldwide are cause for concern considering their contribution to crop productivity through pollination [21–22]. Furthermore, the over reliance on a single bee species for pollination can make the food production system particularly vulnerable, as declining honey bee populations have recently demonstrated [23–24]. As a consequence, strategies that promote diverse wild bee communities in urban landscapes would not only contribute to urban biodiversity but also increase the reliability of pollination services provided to urban agriculture.

The abundance and diversity of bees can be influenced by both site specific and landscape level variables [22, 25–27]. Because bees require both food and nesting resources, both resources can be available within the site itself or present in the surrounding landscape [28]. At the landscape scale, habitat composition strongly impacts bees [29], with increasing natural areas resulting in greater abundance and richness of wild bees [22]. Forest habitats, for example, can be particularly important for providing nesting habitat, while forest trees and ephemeral herbaceous plants offer pollen and nectar resources in the early spring [30]. At the site scale, a number of factors influence the abundance and richness of urban bees including: site size, management, pollution, and connection to the surrounding landscape [31]. Availability of floral resources at the site level is also important in attracting and supporting bees [32–34]. Lawns with flowering plants can increase bee abundance and richness [35–36], while urban prairies support greater pollinator abundance than turf in city parks [3]. At the site scale, plant selection, flower diversity, and floral characteristics such as flower scent and color can also influence pollinators [37–40]. For urban agricultural sites, availability of food and nesting resources throughout the season both within and surrounding the site will be important for attracting and retaining pollinators. To date, however, few studies have focused on identifying the bee communities and pollination services provided to urban agricultural sites.

Understanding how local and landscape variables differentially affect bees and pollination services in urban agricultural sites will be a critical component to developing urban planning policies that advance biodiversity conservation and the provision of ecosystem services in urban landscapes. Our aim in this study was to determine how wild bees and the provision of pollination services were affected by local and landscape variables in urban agricultural sites. We selected 19 urban agricultural sites in the greater Chicago, Illinois, USA metropolitan area with low to high amounts of local floral resources, and along a gradient of moderate to high amounts of impervious cover, to sample bees and measure pollination services. We predicted that: 1) abundance and richness of pollinators would be positively correlated with the amount of local floral resources and negatively correlated with the amount of impervious cover, 2) pollinator community composition would change across sites with low to high levels of local floral resources, and 3) pollination services would increase as local floral resources increased and the amount of impervious cover decreased in the landscape surrounding sites.

## Methods

### Study area

Data were collected in the greater Chicago metropolitan area in Cook County, which is located in northeastern Illinois, USA (41.8781° N, -87.6298° W). Cook County is predominantly an urban area with one large city, Chicago, and a population of 2.6 million. The city of Chicago is characterized by a highly urbanized city center and surrounding suburban areas, both of which support a variety of private and publicly-owned urban agricultural sites. For this study, we selected urban agricultural sites located on privately owned property and within the Chicago Park District. Using community gardens and urban farms across Cook County, we selected 19 gardens and farms that varied in their amount of surrounding impervious cover and supported a range of within site floral resources. Specifically, we selected five sites with ≥20% of the total area planted in flowers, five sites with ≤5% of the total area planted in flowers, and the remaining 9 sites had intermediate ranges (6–19%) of within site floral resources. Sites were also selected along a gradient of impervious cover with 12 sites having >50% impervious cover and 7 sites with <50% impervious cover in the surrounding landscape. Permission from the park district and property owners was obtained prior to sampling for all study sites. Sampling did not involve endangered or protected species.

### Pollinator sampling

Passive sampling and visual observations were used to quantify the bee community at each of our gardens and farms in the summer of 2011. For passive sampling, we used pan traps. A total of four sampling stations were established at each study site with a minimum of 5 meters separating each station. Each sampling station consisted of 3 pan traps (6 oz white plastic bowl, SOLO PB6) with one fluorescent yellow, one fluorescent blue, and one white trap at each station (for a total of 12 traps/site). Each pan trap fit inside a 10.16 cm diameter PVC pipe cut to 2.54 cm in length that was mounted to a bamboo stake using a zip tie, which allowed the pan trap to be adjusted to the height of the surrounding vegetation. Bamboo stakes and attached traps were positioned in a triangular arrangement with each stake separated by 0.3 meters. The location of sampling stations within urban agricultural sites was determined with the help of site managers and community garden coordinators in an effort to minimize disturbance to traps. In community gardens, access to individual garden plots restricted the placement of sampling stations at some sites; however, all stations were positioned 5 meters from an edge or pedestrian walkway, in a sunny location, and outside the drip line of nearby trees. Bees were sampled over a 48-hour period during which pan traps were filled with a soapy water solution. Sites were sampled simultaneously once in June, July, and August during the third week of each month. All bees collected from pan traps were returned to the lab and identified to species.

Visual observations of pollinator visitors to cucumber flowers were recorded during the time that pollination services were being measured in the field (see “Measuring pollination services” below). Visual observations were performed simultaneously at two separate clusters of cucumbers for a 30-minute period (1 hour /site). In July, visual observations were conducted at all sites, but in June, visual observations were performed at only half of the study sites due to an inability to travel to all sites during the sampling period. All bees that contacted anthers or stigmas were counted as visitors. Bees were classified into the following groups: honey bee (Apidae; *Apis mellifera*), bumble bee (Apidae; *Bombus* spp.), black-horned bee (Apidae; *Melissodes bimaculata*), green bees (Halictidae; *Augochlora*, *Augochlorella*, *Agapostemon*), wool carder bee (Megachilidae; *Anthidium*), other Megachilids, small black bees, and large black bees. Because visual observations were conducted only once at some sites, bee visits were summed for each group and results are presented as percent visitation per group.

### Measuring pollination services

Pollination services were measured at each site using potted cucumber plants (*Cucumis sativus* var. Picklebush) the last week of June and July 2011. Cucumber was selected to measure rates of pollination because cucumbers require an insect to transfer pollen from male to female flowers in order to set fruit [41]. Greenhouse grown cucumbers were transported to the field once plants began flowering. Each study site received 8 potted cucumber plants placed randomly at 2 of the 4 sampling stations with 4 plants placed per station. Exclusion bags made from no-see-um netting (Quest Outfitters, Sarasota FL.) were placed over one cucumber plant at each station to exclude pollinators, while the remaining three plants were open to pollinators. Because cucumber plants are monoecious (both male and female flowers on the same plant), we selected plants that had a minimum of two open male and female flowers at the start of each trial. Cucumber plants remained in the field for a 48-hour period, and the number of open female flowers was counted daily. After exposure to pollinators, plants were returned to the greenhouse to allow fruits to mature. A pollination index was calculated for each site as the number of fruits produced on open plants (n = 6) divided by total number of flowers that bloomed over the experiments multiplied by 100, which calculated the percent fruit set for open plants. Percent fruit set was also calculated for control plants, those that excluded pollinators. The percent fruit set for closed plants was then subtracted from the percent fruit set of open plants. Pollination was measured once in June and once in July during the 4^th^ week of the month. No difference in fruit set was found between June and July, so percent fruit set was averaged across sampling dates.

### Characterization of landscape variables

To investigate the effect of bee abundance, richness, and community composition, landscape variation was quantified in geographic information system (GIS) based on the percentage of different land cover classes in the landscape surrounding urban agricultural sites. Land cover and impervious layers for Cook County, Illinois USA were obtained from the 2006 National Land Cover Database (NLCD 30 m resolution) and used to calculate land cover percentages for five classes: impervious cover, forest (deciduous, coniferous, and mixed forest combined), grassland (pasture and grassland combined), water, and urban green space. The urban green space cover class included all areas dominated by turf, such as cemeteries and parks. ArcGIS 10.0 was used to create buffers around the center of each site, and the “Calculate Area” tool determined the proportion of grassland, forest, green space, and water within each buffer using the NLCD land cover layer. The NLCD impervious layer, which rates each cell on a scale of 0–100% impervious cover, was used to calculate percent impervious cover using the “zonal statistics” tool in ArcGIS 10.0. Because the NLCD impervious layer quantifies the percent impervious cover for each cell, as opposed to the land cover layer which uses three general categories (i.e. low, medium, and high impervious cover), the impervious layer was used as the more precise estimate of percent impervious cover surrounding sites. A preliminary analysis of landscape variables was performed at 300 m, 500 m, 1000 m. The strongest response to landscape variables was found for the 500 m scale except for large-bodied bees, which had the strongest response to the 1000 m scale. Using a scale of 500 m to analyze the response of bees to landscape variables is also supported based on the foraging range of many medium and small-sized bees [42–43], which dominated our pan traps. As a result, all analyses were based on landscape variables calculated at the 500 m scale except the response of large-bodied bees, which were analyzed using landscape variables measured at 1000 m.

### Characterization of local site variables

To assess the effect of local habitat variation on bees, we measured a set of variables that could be found within the property boundaries of urban agricultural sites. Local variables included: flower area, flower diversity, vegetable area, and hardscape. Hardscape included all paved areas within the site. Flower and vegetable areas were quantified by measuring the dimensions of all beds within a site containing flowers or vegetables then expressing the variable as a percentage of the total area of the property. Flower beds were defined as intentionally planted areas that contained any combination of flowering annuals, herbaceous perennials, or shrubs. Vegetable beds were defined as planted areas that contained any combination of perennial or annual fruit or vegetable crops. Flower diversity within flower beds was determined by identifying the separate plant species that bloomed during sampling periods. Hardscape was calculated based on all paved areas (patios, driveways, sidewalks) and buildings within the site and expressed as a percentage of the total area of the property. The presence of managed honey bees was recorded for each site as well as site management practices (i.e., organic or conventional). Measurements for all local variables were completed during the 2011 field season, except flower diversity which was measured once in June, July, and August.

### Data analysis: Pollinator community composition

Bee community composition was compared among urban agricultural sites using non-metric multidimensional scaling (NMDS). The abundance of each bee species was averaged for each site across the 2011 season. The similarity between sites was then quantified using the zero-adjusted, Bray-Curtis coefficient, which alleviates multivariate heteroskedasticity when zeros are present for many of the species. The resulting similarity matrix is the basis for creating a NMDS ordination, in which sites are ranked based on their similarity to each other. Sites with similar pollinator communities are placed closer together in ordination space, and as the distance between sites increases, sites become more dissimilar in composition. The relationship between measured landscape variables (e.g., impervious, hardscape, flower area, and flower diversity) and bee community composition was analyzed using environmental vector fitting. NMDS scores from the community ordination, along with the corresponding environmental vectors, indicated most of the variation in bee community composition and most of the association between bee communities and landscape structure was represented along the second NMDS axis, which represented increasing impervious cover. The abundance of each bee species was averaged across sites and correlated with NMDS axis 2 using Spearman’s ρ to show which bee species were negatively and positively correlated with this NMDS axis. NMDS axis 2 values were also used to represent bee community composition in linear regression modeling, described below. NMDS ordinations and environmental vector fitting were performed using the vegan package [44] in R [45].

### Data analysis: Modeling bee abundance, richness, and fruit set

The relationships between landscape variables and bee abundance, richness, community composition, and fruit set were evaluated using a model-selection approach. First, the number of landscape variables used in model selection was reduced. While forest and grassland cover were anticipated to influence bee metrics, both variables had less than 1% cover in the 500 m surrounding study sites. Due to lack of variation across study sites, grassland and forest were not included as explanatory variables in model selection. Impervious cover and open space, the remaining landscape variables, were significantly negatively correlated (Spearman’s Correlation R = -0.86, P < 0.0001). Because impervious cover is an important variable explaining arthropod response to land cover change [46–47], it was retained for use in model selection. Principle Components Analysis (PCA) was used to identify within-site variables correlated with each other [48]. PC1 had positive loading for percent vegetable area and a negative loading for percent flower area, while PC2 had positive loadings for flower diversity and percent hardscape. Thus, the landscape variables used in model selection included: impervious, hardscape, flower area, flower diversity, and management.

Using a model-selection approach, Akaike Information Criteria (AICc) values were calculated for each model from which we quantified AICc differences, ΔAICc [49]. Models with ΔAICc < 2 are considered competing models and strongly supported by the data. From AICc values, we also calculated model weights, *i*, and variable weights [50–51]. Model weights are used to indicate the importance of a model, with higher weights indicating greater model importance. By summing the weights of all models containing a particular variable, the relative importance of each model variable was determined [49,51]. The higher the calculated weight indicates increased variable importance [51]. Bee abundance did not meet the assumptions of normality and was modeled using a Poisson and negative binominal distribution. Abundance data modeled using both a Poisson and negative binominal model were overdispersed, suggesting models were not a good fit for the data.

We hypothesized poor model fit may be the result of combined bee morphology. As a result, we separated bees into genus-level categories by body size and nesting. Large-bodied cavity nesting bees consisted of *Apis mellifera* and *Bombus* species (~10–23 mm in size) and were modeled using a Poisson distribution with landscape variables measured at 1000 m. Due to the fact honey bees are a managed species and can be placed anywhere in the urban landscape, we wanted to ensure honey bees were not driving the patterns observed in our large-bodied bees. Honey bee and bumble bee abundance were analyzed separately, but the same response to landscape variables was observed. As a result, both species were combined for the final analysis. The abundance of small-bodied soil nesting bees was represented by *Halictus* species (~7–11 mm in size) while small-bodied cavity nesting bees included *Hylaeus* species (~4–6 mm in size). Bees in the genera *Halictus* and *Hylaeus* were selected for model selection due to the occurrence of individuals across sites. Abundance data for small-bodied bees was modeled using a negative binomial distribution with landscape variables measured at 500 m. Model assumptions including overdispersion were satisfied when abundance was modeled by body size and nesting. Bee richness, community composition, and fruit set met the assumptions of normality and were modeled using a normal distribution. We also examined R^2^ values to evaluate which models explained the most variation in the data for bee richness, community composition, and fruit set. AICc and R^2^ values were determined in R version 3.0 [45]. Available degrees of freedom prevented the inclusion of interaction terms in our models for fruit set, bee abundance, richness, and community composition. As a result, all variable combinations except interactions were used to construct our model set (7 possible models).

## Results

**Fig 1:**
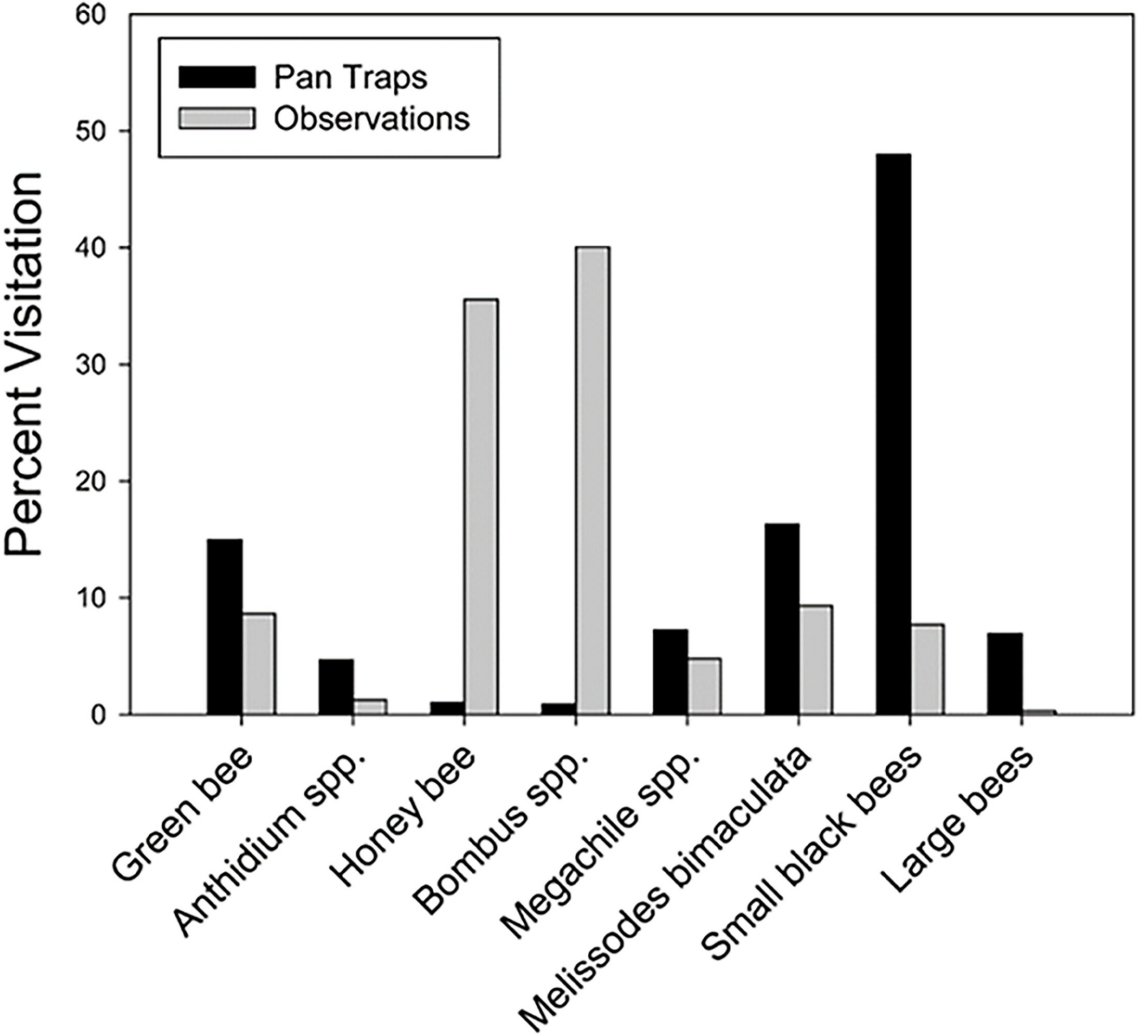
Percent visitation of bee groups. Percent visitation of bee groups were collected at 19 urban agricultural sites using pan traps (black bars) and visually observed (gray bars) visiting cucumber flowers during 2011.

**Fig 2:**
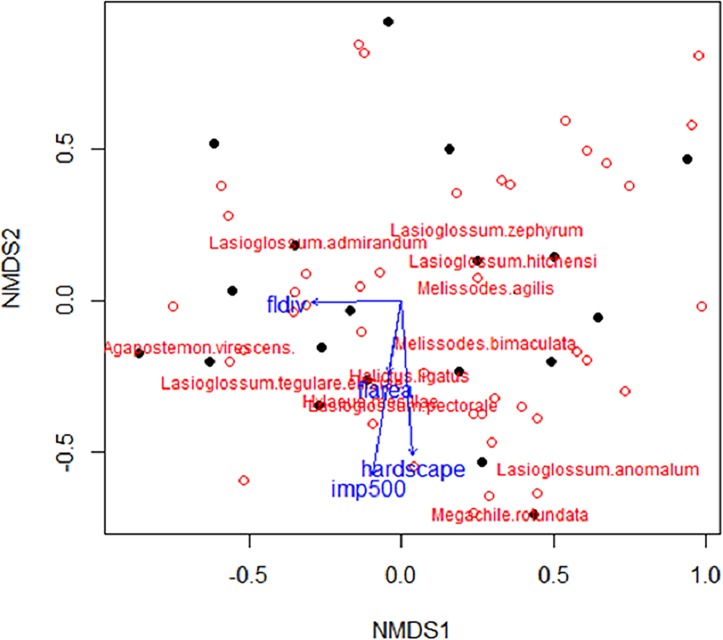
Bee community composition in relation to local and landscape variables. NMDS ordination depicts the relationship between study sites (black points) and landscape variables (blue vectors) in 2-dimensional space. Impervious cover was the only landscape variable significantly negatively correlated with axis 2 (R^2^ = 0.48, *P* = 0.009). No variables were significantly correlated with axis 1. Open circles represent bee species with abundances greater than 35 individuals, which highlights the most common species collected.

A total of 1,384 bees from 75 different species were collected from pan traps during the 2011 field season across 19 urban agricultural sites. The total number of bees collected from individual gardens ranged from 22 to 145 across the three sampling dates. To account for the difference in bee abundance across sites, rarefied bee richness was calculated for each site and ranged from 8.5 to 14.5. During visual observations of cucumber flowers, we observed 1,583 bee visits during 1800 minutes (30 hours) of observation across all study sites. Visual observations indicated bumble bees (*Bombus* spp.) were the most common visitor, composing ~40% of the visits but followed closely by honey bees (*Apis mellifera*), which contributed to ~35% of the bee visits (Fig 1). The NMDS and vector fitting analyses showed that bee community composition changed along a landscape gradient (Fig 2, two-dimensional stress = 0.17), where communities associated with high proportions of impervious cover had negative NMDS axis scores. Along NMDS axis 1, the analysis indicated sites with high within-site flower area had negative NMDS axis scores while sites along NMDS axis 2 with high within-site hardscape had negative NMDS axis scores. Percent impervious, hardscape, and flower area were negatively correlated with the second NMDS axis with only impervious being significantly negatively correlated with axis 2 (Fig 2; Impervious: R^2^ = 0.48, *P* = 0.009; Hardscape: R^2^ = 0.25, *P* = 0.08; Flower area: *P* > 0.05). In contrast, flower diversity was negatively correlated with NMDS axis 1 (Fig 2: Flower diversity: *P* > 0.05). The abundance of bee species averaged by site was correlated with NMDS axis 2 using Spearman’s (ρ) (S1 Table). *Hylaeus leptocephalus*, *Hylaeus punctatus*, *Hylaeus* spp., *Lasioglossum pectorale*, and *Melissodes bimaculata* were all significantly negatively correlated with NMDS axis 2, meaning these bees were increasing in abundance as impervious cover increased. In contrast, *Lasioglossum paradmirandum* and *Lasioglossum perpunctatum* were both significantly positively correlated with NMDS axis 2, meaning these species were increasing in abundance as impervious cover decreased.

### Model selection: Bee abundance, bee richness, community composition, and fruit set

Among all possible models, bee abundance for large-bodied bees, honey bees and bumble bees, was best explained by impervious cover and flower area (Table 1). The overall best model, which included impervious cover and flower area, accounted for 30% of the model weights. Only one competing model was found, which included only impervious cover and accounted for 15% of the model weights. In both models, impervious cover was significantly positively correlated with the abundance of large-bodied *Bombus* and *Apis* bees. Variable weights identified impervious cover, with a weight of 0.79, as the most influential variable explaining large-bodied bee abundance (Table 2). Flower area had the second highest variable weight at 0.58 followed by flower diversity with a weight of 0.20. Hardscape and management had the lowest variable weights both at 0.18 (Table 2).

**Table 1:** Model selection results for bee abundance by body size and nesting habit.

Abundance	IMP^a^	Hardscape	Fldiv^b^	Flarea^c^	Manage^d^	ΔAICc	W
**Large bodied / Cavity**							
*Bombus + Apis 1000*^*e*^							
1. Flarea + Imp	0.033			-0.031		0.0	0.30
2. Imp	0.03					1.37	0.15
**Small bodied / Soil**							
*Halictid 500*^*f*^							
1 Flarea + Imp + Manage	-0.018			0.029	0.62	0.0	.26
2. Flarea + Manage				0.02	0.68	0.77	.18
**Small bodied / Cavity**							
*Hylaeus 500*^*f*^							
1. Hardscape		0.031				0.0	.42

Only competing models (AICc < 2) are reported.

^a.^ Impervious cover (IMP) measured in the 1000 m surrounding urban agricultural site for large-bodied bees (*Bombus* and *Apis*) and 500 m surrounding sites for small-bodied bees (*Halictus* and *Hylaeus*)

^b.^ Flower Diversity (FLdiv) measured as the number of crop and flower species within sites

^c.^ Flower Area (Flarea) measured as the percent cover of flowering herbaceous species within sites

^d.^ Management (Manage) was recorded as conventional or organic for each site

^e.^ Models for large-bodied *Bombus* and *Apis* used a Poisson distribution with IMP measured at 1000 m

^f.^ Models for *Halictus* and *Hylaeus* bees used a negative binominal distribution with IMP measured at 500 m

**Table 2:** Variable weights calculated for each explanatory variable in the full models for abundance.

Bee body size and nesting	IMP^a^	Flower Area	Flower Diversity	Hardscape	Managed
Large, Cavity (*Bombus /Apis*)	**0.79**	0.58	0.20	0.18	0.18
Small, Soil (*Halictus*)	0.59	**0.89**	0.17	0.22	0.70
Small, Cavity (*Hylaeus*)	0.27	0.15	0.15	**0.92**	0.21

Higher variable weights indicate greater importance.

^a.^ Impervious cover (IMP) measured in the 1000 m surrounding study site for large-bodied bees (*Bombus* and *Apis*) and 500 m surrounding sites for small-bodied bees (*Halictus* and *Hylaeus*)

Bee abundance for small-bodied soil nesting bees, *Halictus* spp., was best explained by a model that included flower area, impervious cover, and site management (Table 1). The overall best model accounted for 26% of the model weights. Only one competing model was found, which included flower area and management, and accounted for 18% of the model weights. For the overall best and competing models, impervious cover measured at 500 m was significantly negatively correlated with *Halictus* abundance while flower area and organic management were significantly positively correlated with abundance. Variable weights identified flower area, with a weight of 0.89, as the most influential variable explaining small-bodied soil nesting bees. Site management had the second highest variable weight at 0.7 followed by impervious cover with a weight of 0.59. Hardscape and flower diversity had the lowest variable weights both at 0.22 and 0.17, respectively (Table 2).

Bee abundance for small-bodied cavity nesting bees, *Hylaeus* spp., was best explained by a model that included only hardscape (Table 1). Hardscape was significantly positively correlated with increasing *Hylaeus* abundance. The overall best model accounted for 42% of the model weights, and no competing models were found. Variable weights identified hardscape, with a weight of 0.92, as the most influential variable explaining small-bodied soil nesting bees. Impervious cover and site management had similar variable weights at 0.27 and 0.21, respectively (Table 2). Flower diversity and flower area had the lowest variable weights both at 0.15.

In contrast to abundance, bee richness was best explained by the amount of impervious cover and flower area in the landscape. The overall best model included only impervious cover measured at 500 m, accounted for 19% of the model weights, and explained 14% of the variation in bee richness (Table 3). In all competing models, impervious cover was negatively correlated with bee richness. Although the correlation was not significant, the trend indicated bee richness decreased as impervious cover increased (R^2^ = 0.14, *P* = 0.10). Variable weights for bee richness were 0.52 for impervious cover, 0.28 for flower area, 0.20 for hardscape, 0.18 for management, and 0.17 for flower diversity (Table 4).

**Table 3:** Model selection results for bee richness (rarefied), community composition, and fruit set.

Models	IMP^a^	Hardscape	FLdiv^b^	Flarea^c^	Manage^d^	ΔAICc	R^2^	W
*Richness*								
1 Imp	-0.045					0.0	0.14	0.19
2. Intercept only						0.12	0.0	0.18
3. Imp + Flarea	-0.063			0.044		0.87	0.25	0.12
*Community Composition*								
1. Imp + Hardscape	-0.010	-0.01				0.0	0.48	0.28
2. Imp	-0.013					0.87	0.34	0.18
*Fruit Set*								
1. Hardscape		-0.003				0.0	0.23	0.29

Only competing models (AICc < 2) are reported.

^a.^ Impervious cover (IMP) measured in the 500 m surrounding study site

^b.^ Flower Diversity (FLdiv) measured as the number of crop and flower species blooming within sites

^c.^ Flower Area (Flarea) measured as the percent cover of flowering herbaceous species within sites

^d.^ Management (Manage) was recorded as conventional or organic for each site

**Table 4:** Variable weights calculated for each explanatory variable in the full model for bee richness, community composition, and fruit set.

	Impervious^a^	Hardscape	Flower area	Flower diversity	Management
Richness	**0.52**	0.20	0.28	0.17	0.18
Community Composition	**0.81**	0.60	0.18	0.14	0.20
Fruit Set	0.18	**0.66**	0.24	0.16	0.23

Higher variable weights indicate greater variable importance.

^a^ Impervious cover measured in the 500 m surrounding study sites.

**Fig 3:**
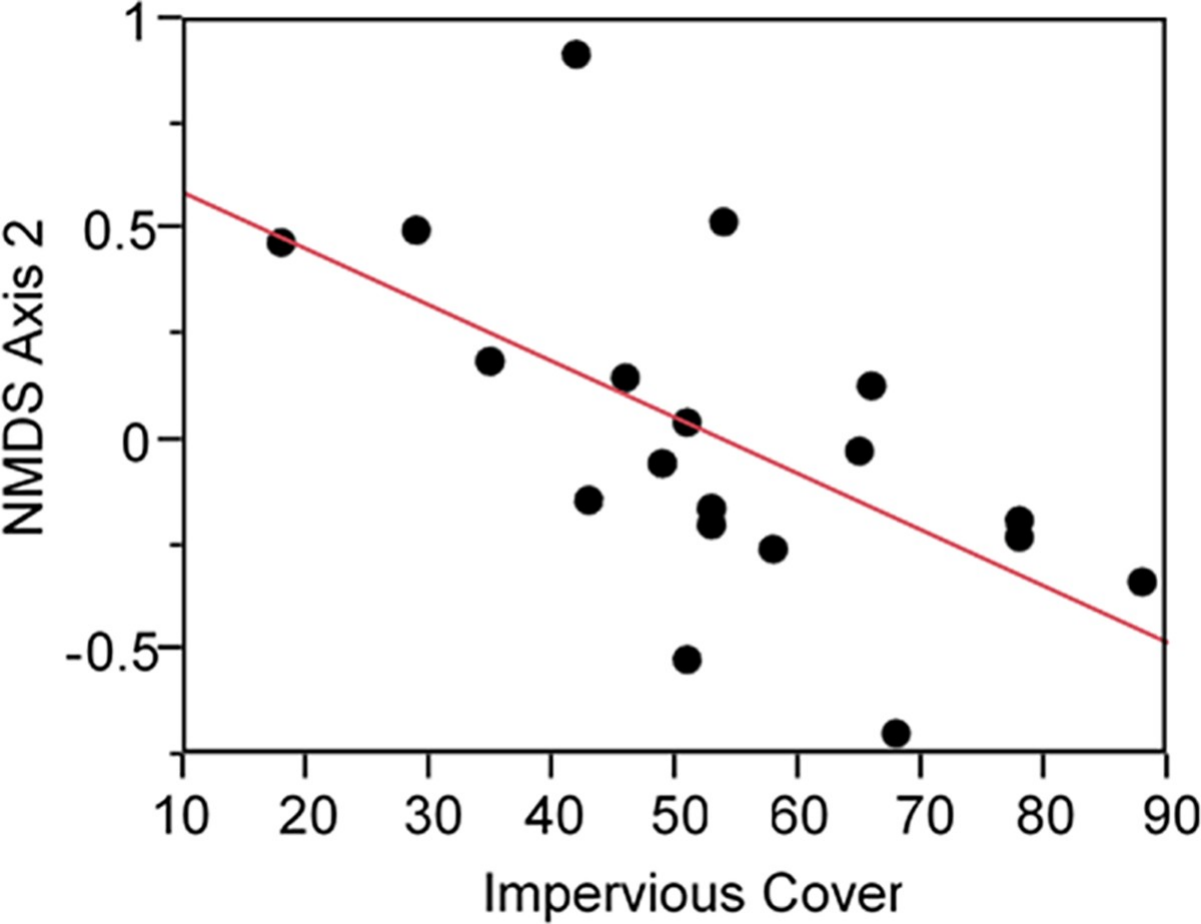
Linear regression showing the relationship between NMDS axis 2 and percent impervious cover. A significant negative correlation was found between bee community composition (NMDS axis 2) and impervious cover measured in the 500 m surrounding study sites (R^2^ = 0.34; *P* = 0.0078).

Bee community composition was best explained by impervious cover and hardscape (Table 3). The overall best model, which included impervious cover and hardscape, accounted for 28% of the model weights and explained 48% of the variation in the data. Only one competing model was found which included only the variable impervious cover, which accounted for 18% of the model weights and explained 34% of the variation in the data. Impervious cover had the highest variable weight at 0.81 indicating this variable was as the most influential variable explaining community composition and had a significant negative relationship with community composition (Table 4; Fig 3, R^2^ = 0.34, *P* = 0.0078). Hardscape had the second highest variable weight at 0.60 followed by management with a weight of 0.20. Flower area and flower diversity had the lowest variable weights at 0.18 and 0.14, respectively.

**Fig 4:**
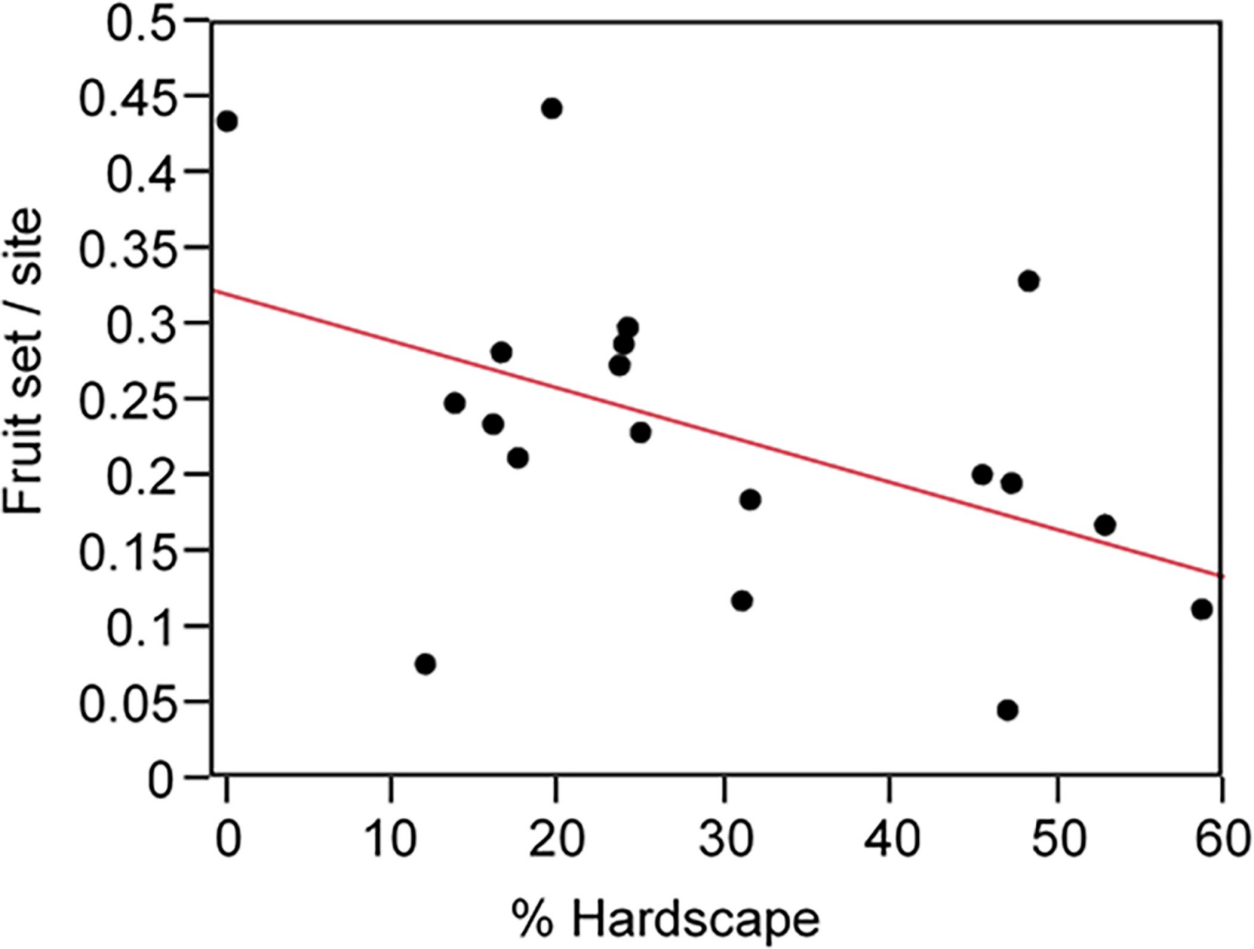
Linear regression showing the relationship between fruit set and percent hardscape. A significant negative correlation was found between fruit set measured using sentinel cucumber plants at each study site and percent hardscape (R^2^ = 0.23; *P* = 0.038).

Model results explaining fruit set included only one model, with hardscape as the only explanatory variable. Hardscape accounted for 29% of the model weights and explained 23% of the variation in fruit set (Table 3). Variable weights indicated hardscape was the most influential variable explaining fruit set with a variable weight of 0.66 (Table 4). The remaining variables had similar weights ranging from 0.24 to 0.16 (Table 4). Fruit set was significantly negatively correlated with hardscape (Fig 4, R^2^ = 0.23, *P* = 0.038).

## Discussion

Urban green space plays a critical role in supporting biodiversity. However, green space habitats are different in terms of their plant diversity, management, and public use. Urban agricultural sites are a growing component of the urban landscape and could potentially serve as a source of biodiversity conservation and ecosystem service provisioning in urban landscapes. Here, we investigated how urban agricultural sites that varied in both surrounding land cover and local site variables differentially supported bees and pollination services. We found the abundance of large-bodied bees, *Bombus* and *Apis* species, were positively associated with increasing amounts of impervious cover, while the abundance of small-bodied cavity nesting *Hylaeus* species increased as the proportion of hardscape, a local variable, increased. In contrast, the abundance of *Halictus* species, small-bodied soil nesting bees, were positively associated with the local site variable flower area. Overall, bee species richness and community composition were negatively correlated with increasing amounts of impervious cover. However, pollination services were most strongly affected by the local variable hardscape, with fruit set decreasing as hardscape increased.

### Bee abundance

Bee abundance was differentially affected by local and landscape variables depending on body size and nesting. Large-bodied bees have large dispersal capabilities [52–54] and often respond more strongly to landscape scale variables [55]. In this study, impervious cover measured at the landscape scale was the most important variable explaining the abundance of large-bodied bees. However, we found this group was positively associated with increasing amounts of impervious cover. While this result seems counterintuitive and contrary to the findings of other studies where bumble bees decline with increasing amounts of impervious cover [46, 56–58], a highly urbanized landscape in combination with nesting biology and management may explain the patterns observed in this study. Both bumble bees and feral honey bees are cavity nesting social species. Increasing amounts of impervious cover may offer more nesting opportunities for both species, explaining the positive correlation with impervious cover. The dispersal range, on average 1.5 km for honey bees and bumble bees [52,59], may allow these bee species to persist in increasingly urbanized areas by exploiting floral resources in the surrounding landscape across scales smaller bodied bees are not capable of searching [60].

The abundance of small-bodied soil nesting bees, composed of *Halictus* species, responded positively to the local variables flower area and organic management but negatively to increasing amounts of impervious cover. *Halictus* species have short foraging distances, ~90–370 m, compared to the larger honey bees and bumble bees [53, 60–61]. Because small-bodied Halictids have shorter foraging distances, we might expect small-bodied bees to be more affected by local variables. While flower area was the most important variable impacting *Halictus* abundance, site management was the second most important variable explaining *Halictus* abundance. We found organically managed sites positively affected *Halictus* abundance while conventional sites negatively affected their abundance. Site management is again a local variable, which could affect both the quality of the food and nesting resources. A set of local variables not evaluated in this study but documented to influence bees are flower scent and color [62–64]. Future studies evaluating the importance of local site design and plant selection for attracting and supporting bees should consider including sensory stimuli in addition to the food and nesting resources within sites. Finally, the landscape variable impervious cover was negatively correlated with the abundance of Halictids. *Halictus* bees are soil nesting [65], meaning increasing amounts of impervious cover in the surrounding landscape combined with a smaller dispersal range could limit nesting resources, explaining the negative impact of impervious cover. Our results highlight the importance of local variables, flower area and management, increasing the abundance of *Halictus* bees through the availability and quality of floral and nesting resources. In contrast, impervious cover likely lowered *Halictus* abundance across the larger urban landscape through reducing soil nesting habitat.

Small-bodied cavity nesting *Hylaeus* species were positively affected by the local variable hardscape. Similar to the small-bodied *Halictus* species, the foraging distance for *Hylaeus* species is relatively small, ~300 m, compared to larger-bodied honey bees and bumble bees [43, 53]. Due to a small foraging range, we again expected this group of bees would be more influenced by local than landscape variables. Here, we found as hardscape increased in cover the abundance of this small-bodied group of cavity nesting bees also increased, suggesting the proportion of hardscape may be linked to more nesting opportunities. Similar increases in cavity nesting bees have been reported for areas with higher amounts of urbanization [66–67]. Both groups of small-bodied bees, *Halictus* and *Hylaeus*, were most strongly affected by local landscape variables, while the most important variable influencing the larger bodied honey bees and bumble bees was the landscape variable, impervious cover. Similar to the trends in our study, research has found bees respond to landscape variables at a scale corresponding to body size [28, 55, 68]. The abundance of both groups of cavity nesting bees, large-bodied honey and bumble bees and small-bodied *Hylaeus*, was positively correlated with increasing amounts of paved surfaces. The difference in the response of these groups being the large-bodied honey and bumble bees responded to increases in impervious cover, a landscape variable, and the small-bodied *Hylaeus* responded to increases in hardscape, a local variable. Increasing amounts of impervious cover and hardscape may represent more nesting opportunities for cavity nesting bees with species responding to variables measured at the appropriate scale for their respective foraging range.

### Bee richness

Bee richness decreased as the amount of impervious cover increased in the 500 m surrounding study sites. While this finding was expected, our research confirms patterns observed in both urban and natural systems of declining bee richness with increasing impervious cover [46, 56, 58, 69]. Loss of bee species richness as impervious cover intensifies towards city centers is likely due to fewer resource rich patches of pollen and nectar coupled with declining nesting opportunities for soil nesting bees [70–71]. While small and medium-sized bees are expected to be impacted more strongly by increasing impervious cover due to their small dispersal ranges, Jha and Kremen [57] found bumble bee nesting density was negatively impacted by the amount of paved surface, suggesting even large-bodied bees can be negatively impacted by paved surfaces. In this study, we found bumble bees were the most frequent visitors to cucumber plants, suggesting impervious cover was not affecting their abundance. We did, however, observe ~90% of the *Bombus* visits were by *Bombus impatiens*. The fact *B*. *impatiens* was the dominate *Bombus* visitor may suggest some bumble bee species are more sensitive to increasing amounts of impervious cover. In addition to the availability of soil nesting habitat being reduced as impervious cover increases, the quality of the existing habitat may be affected in urban areas by soil compaction, contamination, or frequent disturbance. Both the declining quantity and quality of foraging and nesting resources likely contributes to the loss of bee richness associated with increasing impervious cover observed in this study.

### Community composition

Community composition changed as the proportion of impervious cover increased. We found several bee species were significantly correlated with sites surrounded by higher amounts of impervious cover including *Hylaeus leptocephalus*, *Hylaeus punctatus*, an undetermined *Hylaeus* species, *Lasioglossum pectoral*e, and *Melissodes bimaculata*. *Hylaeus* species are cavity nesting generalists, having attributes that may make them adaptable to living in more urbanized environments. While nesting opportunities for soil nesting bees decrease as impervious cover increases and soil often becomes more compacted [71], the availability of cavity nesting sites persists and may increase as impervious cover and the attributes associated with impervious cover such as buildings and homes increase. In contrast to *Hylaeus* species, *M*. *bimaculata* has a larger body size, but similar to *Hylaeus* has a wide host range, visiting over 100 different flower species [72]. The wide host range and larger dispersal capabilities of *Melissodes bimaculata* may allow this bee to persist in more urbanized landscapes. Research of urban bees has found *M*. *bimaculata* in New York City gardens, and a frequent visitor to cucumber plants in Chicago gardens [19, 73]. Research on bee community composition also found bee communities in urban, agricultural, and forested sites were significantly different with small-bodied bees in the genus *Lasioglossum* dominating urban areas [74]. *Lasioglossum pectorale* is a smaller bodied soil nesting bee, which makes this bee seem an unlikely candidate for successfully exploiting urban environments. Despite requiring soil nesting habitat, this bee also has a wide host range visiting over 100 recorded flowers including commonly planted ornamental trees and shrubs such as *Prunus*, *Crataegus*, *Potentilla*, and rose as well as weedy species including asters, chicory, goldenrods, mustards and clovers [75], which may help this bee successfully exploit urban landscapes.

Two bee species, *L*. *paradmirandum* and *L*. *perpunctatum*, were significantly correlated with lower levels of impervious cover. Both *L*. *paradmirandum* and *L*. *perpunctatum* are smaller bodied soil nesting bees [65, 76]. While L. *perpunctatum* is considered common with a broad host range, *L*. *paradmirandum* has a narrower host range visiting flowers from only two plant families Asteraceae and Salicaceae [76–77]. Suitable nesting sites as well as floral resources in the aster and willow family are likely to increase in abundance as natural areas and parks increase with decreasing impervious cover. The nesting biology and dietary requirements of these two *Lasioglossum* bees may explain why they were present in bee communities associated with lower amounts of impervious cover.

### Abundance, visual observations

Visual observations determined that bumble bees and honey bees were the most frequent visitors to cucumber plants. Because visual observations were only conducted during the two trials that measured pollination services, our data set is small. Despite this limitation, the visual observations provided valuable information regarding which bee species were actually visiting cucumber flowers and potentially contributing to pollination services as opposed to the information collected from pan traps, which only indicated which bees were present in urban agricultural sites. Data collected from pan traps and visual observations were quite different. Pan traps suggested small black bees were the most common bee occurring at study sites, but visual observations indicated bumble bees and honey bees collectively provided ~67% of the visits to cucumber flowers. Differences in bee data between pan traps and visual observations is not surprising as other studies have found contrasting results depending on the sampling method employed [78–79]. The use of both sampling techniques is recommended because different species are sampled more effectively using each method, which results in less bias and a more comprehensive understanding of the bees present at a particular site [78]. In this study, pan traps indicated small black bees such as *Halictus* and *Lasioglossum* species were the most common, but visual observations indicated bumble bees and honey bees were likely providing the pollination services to sentinel cucumber plants.

### Fruit set

Model selection found hardscape was the most important variable explaining fruit set, and fruit set was significantly reduced as hardscape increased. As within site hardscape increases, the area remaining for floral resource plantings and soil nesting sites decreases. Our results demonstrated the abundance of soil nesting *Halictus* bees and overall bee richness were negatively affected by local site factors such as management and loss of flower area. While small and medium-sized solitary bees were not the primary visitors to cucumber flowers, their decline at sites with higher amounts of hardscape likely contributed to the negative correlation between hardscape and fruit set. Despite a significant effect of hardscape on fruit set, hardscape explained a relatively small amount, ~23%, of the variation in the data, suggesting other variables not captured in our dataset were contributing to differences in pollination across sites.

Visual observations of cucumber flowers indicated honey bees and bumble bees were the dominant visitor. Collectively, honey bees and bumble bees contributed to 67% of the visits to flowers compared to smaller bees which composed only 15% of the visits. While visitation does not ensure pollination, both honey bees and bumble bees are documented visitors to cucumbers and considered important pollinators [41, 80–81]. Honey bees and bumble bees may be one variable not captured in model selection that is contributing to cucumber pollination across study sites. Furthermore, honey bees are a managed species, meaning hives can be placed and maintained even in highly urbanized areas. In Chicago, managed honey bee hives are common, and hive locations are registered with the state of Illinois. We found registered honey bee hives were placed within the average flight range (1500 m) of all but four study sites (S1 Fig), suggesting honey bees may be providing pollination services to gardens and farms. While model selection did find fruit set decreased with increasing hardscape suggesting some pollinators likely small-bodied bees were contributing to pollination, visual observations indicated the primary pollinators of cucumber were honey bees and bumble bees, suggesting a few generalist pollinators may be responsible for providing the majority of pollination services to cucumber plants. The provision of pollination services across urban agricultural sites with some farms located in highly urbanized areas is encouraging. However, pollination services that rely on only a few bee species to provide pollination are highly susceptible to the loss of a primary pollinator. Developing local site management and landscape scale urban planning strategies that support diverse pollinator communities will be critical to providing reliable pollination services to urban agricultural sites.

### Implications for site design and urban planning

Improving native bee conservation and the provision of subsequent pollination services to urban agricultural sites will require management strategies focused at two scales: the site level and the city scale. Here, we found bees responded differently to local and landscape variables depending on body size and nesting habits. Our results demonstrated small-bodied *Halictus* bees benefited from increasing amounts of local floral resources, suggesting the foraging limitations of small-bodied bees should be considered in local site designs. One potential design solution urban agricultural sites could implement is incorporating native flower species around the perimeter of the urban agricultural site or between vegetable beds to increase food and nesting resources as well as facilitate dispersal across the site. This design solution could improve the provision of pollination services as well as other ecosystem services such as natural pest suppression and site aesthetics. Our results also demonstrated some small and large-bodied cavity nesting bees can persist and potentially benefit from novel nesting opportunities created as impervious cover increases. While the management implications of this finding are encouraging and indicate urban agriculture can be successful even in highly urbanized landscapes, our results also found overall bee richness declined with increasing impervious cover. Based on these results, we advocate for urban planning strategies at the city scale that strive to maximize overall bee richness. For example, an urban planning strategy that focuses on reducing the proportion of impervious cover and increasing diverse green space habitat would increase food resources and create natural nesting opportunities for both cavity and soil nesting bee species. Urban planning policies that require commercial and residential sites to limit within site hardscape is one strategy for reducing impervious cover across a city. Alternatively, urban planning strategies that incorporate diverse habitats such as prairies, woodlands, and urban farms into existing parks, vacant lots, and rooftops would increase plant diversity and promote pollinator conservation at a regional scale. Urban planning strategies that conserve pollinators at the city scale in turn create an opportunity for urban farmers to attract and augment bees locally through site design and management practices. More research is needed to better understand the contributions of nearby green spaces present at intermediate spatial scales such as street plantings, residential yards, and rooftop gardens for supporting food and nesting resources. However, this study does provide early evidence that local site design coupled with regional planning can advance pollinator conservation and benefit urban agriculture by creating opportunities for sustainable farming, which is increasingly important as public demand and policy shifts towards locally grown and environmentally responsible food production.

## Supporting information

S1 Fig Location of urban agricultural sites and registered honey bee hives across the city of Chicago. Location of 19 urban agricultural sites^a^, white circles, sampled during the 2011 field season across the city of Chicago. The location of registered honey bee hives, blue circles, in the City of Chicago with a 1.5 km buffer around the location of the hive shows the average flight range of the honey bees in relation to sampled urban agricultural sites.
^a^ The location of two urban agricultural sites in one location are in close proximity to each other and at the scale of this map appear as one site. As a result, the map shows only 18 sites with two overlapping points.
(TIF)

S1 Table Abundance of bee species averaged by site and correlated (Spearman’s ρ) with NMDS axis 2. (PDF)
