# Supplementary figures and images for: Landscape and local site variables differentially influence pollinators and pollination services in urban agricultural sites
Source: PLoS One. 2019 Feb 13;14(2):e0212034. doi: 10.1371/journal.pone.0212034 (PMC6373950; doi:10.1371/journal.pone.0212034)

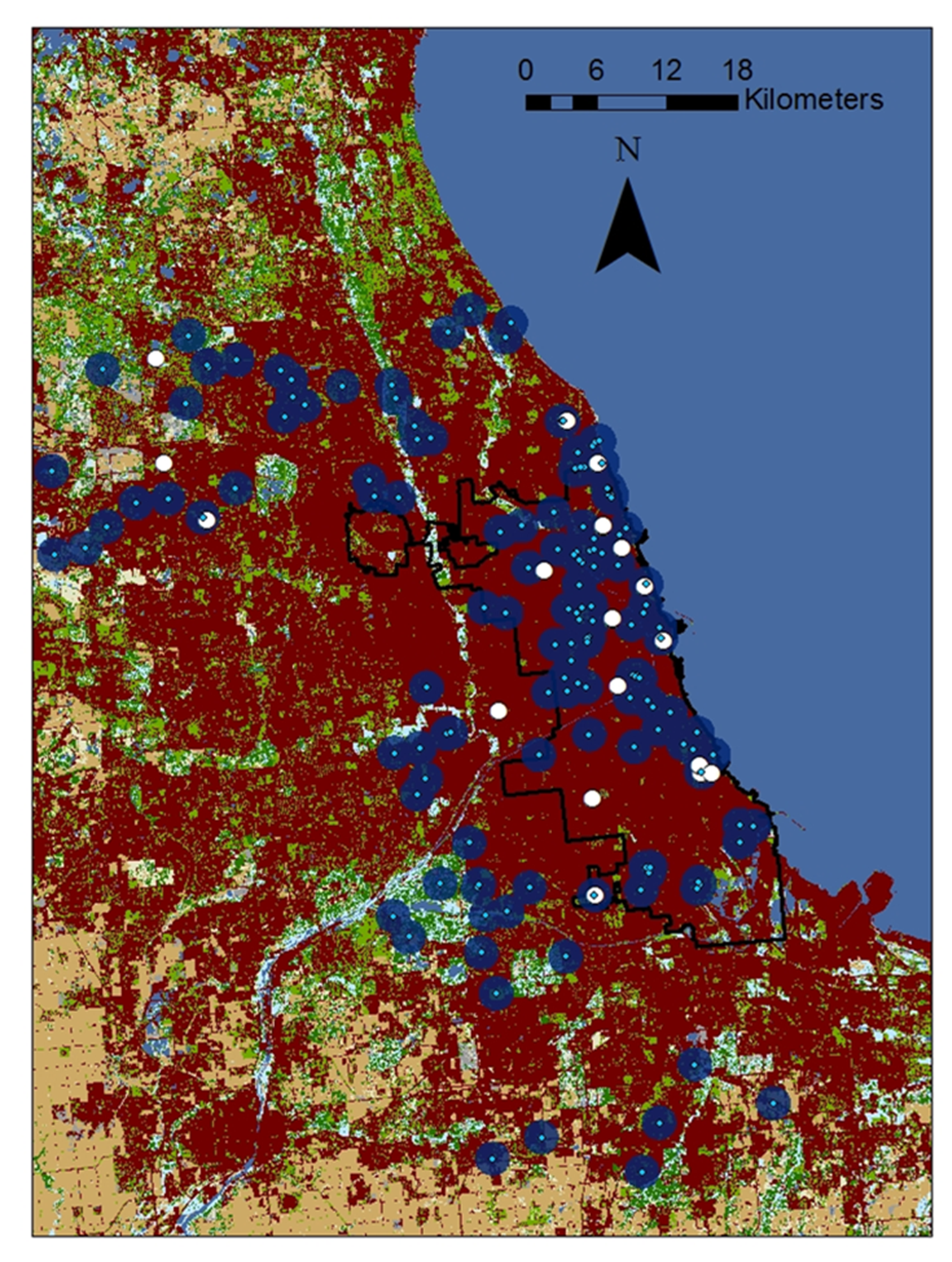

Supplement: S1 Fig — Location of 19 urban agricultural sitesa, white circles, sampled during the 2011 field season across the city of Chicago. The location of registered honey bee hives, blue circles, in the City of Chicago with a 1.5 km buffer around the location of the hive shows the average flight range of the honey bees in relation to sampled urban agricultural sites. a The location of two urban agricultural sites in one location are in close proximity to each other and at the scale of this map appear as one site. As a result, the map shows only 18 sites with two overlapping points. (TIF) [file pone.0212034.s001.tif]
