# Supplementary material for: Landscape and local site variables differentially influence pollinators and pollination services in urban agricultural sites
Source: PLoS One. 2019 Feb 13;14(2):e0212034. doi: 10.1371/journal.pone.0212034 (PMC6373950; doi:10.1371/journal.pone.0212034)
Supplement: S1 Table — (PDF) [file pone.0212034.s002.pdf]

S1 Table. Abundance of bee species averaged by site and correlated (Spearman's ρ) with NMDS axis 2.

Bee Species	Spearman ρ[a]	*P*
Agapostemon virescens	-0.3397	0.1547
Andrena mariae	0.2582	0.2858
Andrena wilkella	-0.3873	0.1014
Anthidium manicatum	-0.4099	0.0813
Anthidium oblongatum	0.0302	0.9022
Anthophora terminalis	-0.1291	0.5984
Apis mellifera	0.0514	0.8346
Augochlora pura	0.2914	0.2262
Augochlorella aurata	0.1974	0.4178
Augochlorella persimilis	0.1777	0.4667
Bombus bimaculatus	0	1
Bombus fervidus	-0.1291	0.5984
Bombus griseocollis	-0.3455	0.1473
Bombus impatiens	0.2378	0.327
Calliopsis andreniformis	-0.2322	0.3387
Ceratina calcarata	-0.0861	0.7261
Certina complex	0.0296	0.9042
Ceratina strenua	0.0498	0.8397
Chelostoma rapunculi	-0.4201	0.0733
Halictus confusus	0.473*	0.0408
Halictus ligatus	-0.3886	0.1001
Halictus rubicundus	0.3131	0.1918
Hoplitis producta	-0.1477	0.5462
Hylaeus fedorica	-0.3443	0.1489
Hylaeus hyalinatus	-0.0198	0.9359

Hylaeus leptocephalus	-0.4811*	0.037
Hylaeus mesillae	-0.0963	0.695
Hylaeus sp.	-0.4751*	0.0398
Hylaeus punctatus	-0.646**	0.0028
Lasioglossum admirandum	0.1895	0.4371
Lasioglossum anomalum	0.1357	0.5797
Lasioglossum cinctipes	0.4093	0.0819
Lasioglossum coeruleum	0.1879	0.4412
Lasioglossum coriaceum	0.1699	0.4869
Lasioglossum cressonii	-0.1949	0.4239
Lasioglossum divergens	0.1942	0.4257
Lasioglossum hitchensi	0.1382	0.5727
Lasioglossum illinoense	0.0626	0.799
Lasioglossum mitchelli	0.24	0.3222
Lasioglossum oblongum	0.2582	0.2858
Lasioglossum oenotherae	0.043	0.8611
Lasioglossum paradmirandum	0.4847*	0.0354
Lasioglossum pectinatum	0	1
Lasioglossum pectorale	-0.5868**	0.0083
Lasioglossum perpunctatum	0.5765**	0.0098
Lasioglossum pilosum	-0.1903	0.4353
Lasioglossum platyparium	0.3873	0.1014
Lasioglossum pruinosum	0.1681	0.4915
Lasioglossum laevissimum/smilacinae	0.2556	0.2908
Lasioglossum sp.	-0.1318	0.5908
Lasioglossum tegulare/ellisiae	-0.3785	0.1101
Lasioglossium weemsi	0.1721	0.481

Lasioglossum zephyrum	0.2044	0.4012
Megachile campanulae	0.3012	0.2101
Megachile centuncularis	-0.1566	0.5221
Megachile concinna	-0.3012	0.2101
Megachile inimica	-0.3443	0.1489
Megachile latimanus	-0.3443	0.1489
Megachile mendica	-0.1648	0.5002
Megachile pugnata	-0.3689	0.1201
Megachile rotundata	-0.3761	0.1125
Megachile sculpturalis	0.3012	0.2101
Megachile texana	-0.3443	0.1489
Melissodes agilis	-0.1642	0.5018
Melissodes bimaculata	-0.5007*	0.029
Melissodes denticulatus	-0.2152	0.3764
Melissodes desponsus	-0.0041	0.9866
Melissodes illatus	-0.0724	0.7683
Melissodes sp.	0.3012	0.2101
Melissodes subillatus	-0.236	0.3308
Melissodes trinodis	-0.3873	0.1014
Peponapis pruinosa	0.3377	0.1574
Stelis coarctatus	0.043	0.8611
Stelis lateralis	0.2582	0.2858
Xylocopa virginica	0.3953	0.0939

[a] A correlation significant at <0.05 is indicated by one asterisk,*, while a correlation significant at <0.01 is indicated by two asterisks, **.
